# Obesity Disparities Among Adult Single-Race and Multiracial Asian and Pacific Islander Populations

**DOI:** 10.1001/jamanetworkopen.2024.0734

**Published:** 2024-03-19

**Authors:** Adrian M. Bacong, Sophia L. Gibbs, A. Gabriela Rosales, Timothy B. Frankland, Jiang Li, Yihe G. Daida, Stephen P. Fortmann, Latha Palaniappan

**Affiliations:** 1 Department of Medicine, Division of Cardiovascular Medicine, Stanford University School of Medicine, Stanford, California; 2 Stanford Center for Asian Health Research and Education, Stanford, California; 3 University of Minnesota Medical School, Minneapolis; 4 Kaiser Permanente Center for Health Research, Portland, Oregon; 5 Center for Integrated Health Care Research, Kaiser Permanente Hawai’i, Honolulu; 6 Sutter Health Center for Health Systems Research/Palo Alto Medical Foundation Research Institute, Palo Alto, California

## Abstract

**Importance:**

Despite increasing numbers of multiracial individuals, they are often excluded in studies or aggregated within larger race and ethnicity groups due to small sample sizes.

**Objective:**

To examine disparities in the prevalence of obesity among single-race and multiracial Asian and Pacific Islander individuals compared with non-Hispanic White (hereafter, White) individuals.

**Design, Setting, and Participants:**

This cross-sectional study used electronic health record (EHR) data linked to social determinants of health and health behavior data for adult (age ≥18 years) members of 2 large health care systems in California and Hawai’i who had at least 1 ambulatory visit to a primary care practitioner between January 1, 2006, and December 31, 2018. Data were analyzed from October 31, 2022, to July 31, 2023.

**Exposure:**

Self-identified race and ethnicity provided in the EHR as a single-race category (Asian Indian, Chinese, Filipino, Japanese, Native Hawaiian only, Other Pacific Islander, or White) or a multiracial category (Asian and Pacific Islander; Asian, Pacific Islander, and White; Asian and White; or Pacific Islander and White).

**Main Outcomes and Measures:**

The main outcome was obesity (body mass index [BMI] ≥30.0), based on last measured height and weight from the EHR. Logistic regression was used to examine the association between race and ethnicity and odds of obesity.

**Results:**

A total of 5229 individuals (3055 [58.4%] male; mean [SD] age, 70.73 [11.51] years) were examined, of whom 444 (8.5%) were Asian Indian; 1091 (20.9%), Chinese; 483 (9.2%), Filipino; 666 (12.7%), Japanese; 91 (1.7%), Native Hawaiian; 95 (1.8%), Other Pacific Islander; and 888 (17.0%), White. The percentages of individuals who identified as multiracial were as follows: 417 (8.0%) were Asian and Pacific Islander; 392 (7.5%), Asian, Pacific Islander, and White; 248 (4.7%), Asian and White; and 414 (7.9%), Pacific Islander and White. A total of 1333 participants (25.5%) were classified as having obesity based on standard BMI criteria. Obesity was highest among people who identified as Asian, Pacific Islander, and White (204 of 392 [52.0%]) followed by those who identified as Other Pacific Islander (47 of 95 [49.5%]), Native Hawaiian (44 of 91 [48.4%]), and Pacific Islander and White (186 of 414 [44.9%]). After accounting for demographic, socioeconomic, and health behavior factors, people who identified as Asian, Pacific Islander, and White (odds ratio [OR], 1.80; 95% CI, 1.37-2.38) or Pacific Islander and White (OR, 1.55; 95% CI, 1.18-2.04) had increased odds of obesity compared with White individuals. All single-race Asian groups had lower odds of obesity compared with White individuals: Asian Indian (OR, 0.29; 95% CI, 0.20-0.40), Chinese (OR, 0.22; 95% CI, 0.17-0.29), Filipino (OR, 0.46; 95% CI, 0.35-0.62), and Japanese (OR, 0.38, 95% CI, 0.29-0.50).

**Conclusions and Relevance:**

In this study, multiracial Asian and Pacific Islander individuals had an increased prevalence of obesity compared with many of their single-race counterparts. As the number of multiracial individuals increases, it will be important for clinical and public health systems to track disparities in these populations.

## Key Points

**Question:**

What is the prevalence of obesity among disaggregated single-race and multiracial Asian and Pacific Islander individuals in the US?

**Findings:**

In this cross-sectional study of 5229 individuals, prevalence of obesity was highest among people who identified as Asian, Pacific Islander, and White (52.0%).

**Meaning:**

The findings suggest that multiracial people are an important group for which to address disparities in obesity and chronic disease.

## Teaser

This cross-sectional study investigates the prevalence of obesity among disaggregated single-race and multiracial Asian and Pacific Islander populations compared with non-Hispanic White individuals in the US.

## Introduction

Cardiovascular diseases remain the leading causes of death both in the US and worldwide.^1,2^ Multiple demographic, social, socioeconomic, behavioral, and clinical factors^1,3^ contribute to cardiovascular disease, including age, sex, diet, exercise, and obesity.^4^ Obesity, or excess body weight, is an important risk factor for cardiovascular disease.^4,5^ The prevalence of obesity (ie, body mass index [BMI]≥30 [calculated as weight in kilograms divided by height in meters squared]) in the US increased from 30.5% in 1999 to 41.9% in 2017.^6^ Obesity prevalence differs by race and ethnicity in the US and is highest among Black individuals (39.7%) and lowest among Asian individuals (29.4%).^7^ However, within Asian and Asian American populations, there is also heterogeneity by racial and ethnic subgroups. Obesity, based on standard cutoffs, was found to be highest among Filipino individuals (15.3%) and lowest among Vietnamese individuals (6.3%).^7^

Although the obesity rate is lower among Asian American populations compared with other racial and ethnic groups, Asian American individuals have higher prevalence of type 2 diabetes^2,8^ and cardiovascular disease.^2^ Furthermore, proportional mortality related to cardiovascular disease^9,10,11,12^ and diabetes^13^ is higher among Asian American individuals compared with other racial and ethnic groups. In comparison, Native Hawaiian and Pacific Islander individuals, who are often aggregated with Asian individuals, have high obesity prevalence. Estimates suggest that 51.7% of Native Hawaiian and Pacific Islander people have obesity.^14^ Furthermore, the prevalence of obesity-related chronic diseases, such as cardiovascular disease and diabetes, is higher among Native Hawaiian and Pacific Islander people compared with Asian people and other racial and ethnic groups.^15,16,17^

Racial differences in obesity may be explained by various demographic, socioeconomic, and health behavior factors. Differences in age and sex composition^7^ may confound the association between race and ethnicity and obesity. Additionally, some Asian subgroups may have higher socioeconomic attainment compared with others,^18^ allowing them to have healthier lifestyles and better access to health care. Some studies have shown differences in health behaviors such as sleep, exercise, and diet. In studies using community samples, Asian individuals typically had shorter sleep duration and poorer sleep quality compared with non-Hispanic White people.^19,20^ Additionally, state-level studies have shown that more Pacific Islander individuals tend to meet physical activity recommendations compared with Asian and non-Hispanic White people.^17^ Moreover, some Asian and Pacific Islander subgroups may have a more Westernized diet (high in fat and carbohydrates) compared with others.^2^

Although there have been efforts to disaggregate Asian American, Native Hawaiian, and Pacific Islander groups in health data, multiracial Asian and Pacific Islander individuals have been less well studied despite the growing population. As of the 2020 census, 17.1% of Asian American people (4.1 million) and 56.6% of Native Hawaiian and Pacific Islander people (900 000) identified as multiracial.^21^ In state and national datasets, people who identify as multiracial are often grouped within an “other” category (eg, other Asian),^8^ sorted within the focal racial and ethnic group of the investigator’s choice (eg, Native Hawaiian and Pacific Islander),^22^ or excluded entirely due to small sample sizes.^7,8^ Given the heterogeneity in obesity and cardiometabolic risk among Asian and Pacific Islander people, it is possible that these risk profiles may also be different among multiracial individuals. In this study, we (1) describe the burden of obesity among disaggregated single-race and multiracial Asian and Pacific Islander people and (2) evaluate the extent to which demographic, socioeconomic, and health behavior factors are associated with differences in obesity by race and ethnicity.

## Methods

### Dataset

In this cross-sectional study, we used electronic health records (EHRs) linked with survey data from the Cardiovascular Disease Among Asians and Pacific Islanders (CASPER) study.^15^ The parent study was approved by the institutional review boards of the Palo Alto Medical Foundation and Kaiser Permanente Hawai’i. The institutional review boards waived informed consent and authorization from EHR participants given that data were scrubbed of personal identifiers. However, sending back completed surveys implied consent to participate for all survey participants who were sampled from the EHR population. This study followed the Strengthening the Reporting of Observational Studies in Epidemiology (STROBE) reporting guideline.^23^

The EHR data were queried from 2 health care systems, one in California (Sutter Health) and the other in Hawai’i (Kaiser Permanente Hawai’i). Records were collected for adults aged 18 years or older who made at least 1 ambulatory visit to a primary care practitioner during the study period (January 1, 2006, to December 31, 2018). Primary care practitioners included family practice, internal medicine, and obstetric/gynecologic practitioners (when participants indicated that their obstetrician/gynecologist was their primary care practitioner). In addition to EHR data, surveys were collected via mail and online from participants aged 40 years or older. These surveys asked for additional information that was unavailable in the EHR, such as diet and exercise behaviors. Survey participants were chosen by case-control matching based on cardiovascular disease presence recorded in the EHR followed by frequency matching based on age, sex, and race and ethnicity. In the survey sampling process, race and ethnicity groups other than non-Hispanic White (hereafter, White) were oversampled to ensure sufficient sample sizes to run analyses. Additional information on the survey sample is provided in the eMethods in Supplement 1. We identified participants with valid survey responses and added their EHR data to the final dataset. Additional information on the sampling strategy for the survey in addition to recruitment and response distributions is available in the eMethods and eFigures 1 and 2 in Supplement 1.

### Outcome

Obesity, defined as a BMI of 30 or greater,^24^ was our primary outcome of interest. We calculated BMI from measurements in the EHR data.

### Independent Variable

Self-identified race and ethnicity was our primary independent variable and was coded from the EHR data based on patients’ responses. We examined the following disaggregated Asian or Pacific Islander groups: Asian Indian, Chinese, Filipino, Japanese, Native Hawaiian only, and Other Pacific Islander. Other Pacific Islander included less populous Pacific Islander groups, such as CHamorro/CHamoru, Fijian, Marshallese, Samoan, Tahitian, and Tongan individuals. Multiracial categories included Asian and Pacific Islander; Asian, Pacific Islander, and White; Asian and White; and Pacific Islander and White. The Asian and Pacific Islander group does not refer to the classic aggregated category of Asian or Pacific Islander but to people who identified with at least 1 Pacific Islander group (eg, Samoan) and 1 Asian group (eg, Japanese). Due to limitations in sample size, we were unable to examine other disaggregated Asian groups (eg, Korean, Vietnamese) and Pacific Islander groups (eg, Marshallese, Tongan).

### Covariates

We considered multiple demographic, socioeconomic, and behavioral factors that may be associated with differences in obesity by race and ethnicity. Demographic factors included age group (40-59, 60-79, or ≥80 years) and sex (female, male) and were derived from the EHR. Socioeconomic factors in the survey included educational attainment (less than high school, high school diploma or General Educational Development, some college, or college degree or above) and yearly income (<$20 000, $20 000-$49 999, $50 000-$150 000, or >$150 000). In addition, behavioral factors in the survey included hours of sleep per night (<6, 6-8, or >8), physical activity, and diet. Physical activity was measured via the International Physical Activity Questionnaire,^25^ which has been previously validated in Asian and Pacific Islander communities.^26^ Physical activity level was dichotomized as underactive (less than 60 minutes of strenuous or 150 minutes of moderate to vigorous activity per week) or active (at least 60 minutes of strenuous or 150 minutes of moderate to vigorous activity per week). Diet was measured using the Rapid Eating Assessment for Participants–Shortened Version (REAP-S),^27^ a series of 13 items asking about the frequency of specific food use—for example, whether participants drank 16 ounces or more of nondiet soda, fruit drink, or punch per day. Frequency was rated as usually or often (1), sometimes (2), rarely (3), and never (4). The 13 items were summed to create a scale ranging from 4 to 52, in which higher scores on the REAP-S indicated a healthier diet.

### Statistical Analysis

We first examined the distribution of obesity and demographic, socioeconomic, and health behavior factors by race and ethnicity. Comparisons of statistical significance were done using a χ^2^ test for categorical variables and analysis of variance for continuous variables after testing for normality. We then used logistic regression to examine the association between race and ethnicity and the odds of obesity with 4 models. Single-race White individuals were used as the reference group in all analyses, as this is the largest racial and ethnic group in the US. Model 1 examined the unadjusted association between race and ethnicity and odds of obesity. Model 2 accounted for both age and sex. Model 3 additionally adjusted for educational level and income. Finally, model 4 built on model 3 and included sleep duration, physical activity, and diet. All analyses used multivariate imputation by chained equations^28^ with 5 iterations to address missingness on key variables using the mice package^29^ in R Studio, version 4.2.2 (R Project for Statistical Computing).^30^ Missingness was generally low (<10%) for all covariates and our outcome except for income (21.5% missing). All statistical analyses were 2-sided hypothesis tests with a significance level of *P* < .05. Data were analyzed from October 31, 2022, to July 31, 2023.

We conducted supplemental analyses of the full EHR data for adults aged 40 years or older with complete race and ethnicity data. We first examined the general distribution of obesity among the EHR sample by race and ethnicity. We then examined the age-and sex-adjusted association of the odds of obesity by race and ethnicity among all EHR participants within this age group. We also examined obesity prevalence with an Asian-specific BMI cutoff^31^ of 27.5 for single-race Asian groups in both EHR and survey-linked data.

## Results

From a total of 855 013 EHR records collected, 13 911 people were sent surveys, with completion by 5229 individuals (37.6%; 2174 [41.6%] female; 3055 [58.4%] male; mean [SD] age, 70.73 [11.51] years). The percentages of single-race participants were as follows: 444 (8.5%) were Asian Indian; 1091 (20.9%), Chinese; 483 (9.2%), Filipino; 666 (12.7%), Japanese; 91 (1.7%), Native Hawaiian only; 95 [1.8%], Other Pacific Islander; and 888 (17.0%), White. The percentages of multiracial participants were as follows: 417 (8.0%) were Asian and Pacific Islander; 392 (7.5%), Asian, Pacific Islander, and White; 248 (4.7%), Asian and White; and 414 (7.9%), Pacific Islander and White. Most participants (3042 [58.2%]) were aged 60 to 79 years, 2521 (48.2%) had at least a college education, and 2292 (43.8%) reported an annual income of $50 000 to $150 000 (Table 1). Participants typically had 6 to 8 hours of sleep per night (3927 [75.1%]) and were underactive (4806 [91.9%]); mean (SD) REAP-S score was 33.12 (6.24). A total of 1333 participants (25.5%) were classified as having obesity based on standard BMI cutoffs.

**Table 1.** Clinical, Demographic, and Behavioral Characteristics of Participants in the Cardiovascular Disease Among Asians and Pacific Islanders Study

Characteristic	Participants^a^												*P* value
	Total (N = 5229)	Asian Indian (n = 444)	Chinese (n = 1091)	Filipino (n = 483)	Japanese (n = 666)	Native Hawaiian only (n = 91)	Other PI (n = 95)^b^	Asian and White (n = 248)	Asian and PI (n = 417)	Asian, PI, and White (n = 392)	PI and White (n = 414)	White (n = 888)	
**Clinical outcomes**													
Obesity per WHO standard	1333 (25.5)	56 (12.6)	93 (8.5)	105 (21.7)	101 (15.2)	44 (48.4)	47 (49.5)	91 (36.7)	176 (42.2)	204 (52.0)	186 (44.9)	230 (25.9)	<.001
WHO weight category^c^													
Underweight	124 (2.4)	7 (1.6)	37 (3.4)	15 (3.1)	29 (4.4)	2 (2.2)	1 (1.1)	4 (1.6)	7 (1.7)	4 (1.0)	8 (1.9)	10 (1.1)	<.001
Healthy weight	1836 (35.1)	191 (43.0)	571 (52.3)	173 (35.8)	287 (43.1)	19 (20.9)	15 (15.8)	65 (26.2)	82 (19.7)	62 (15.8)	91 (22.0)	280 (31.5)	
Overweight	1936 (37.0)	190 (42.8)	390 (35.7)	190 (39.3)	249 (37.4)	26 (28.6)	32 (33.7)	88 (35.5)	152 (36.5)	122 (31.1)	129 (31.2)	368 (41.4)	
Obesity class 1	802 (15.3)	31 (7.0)	68 (6.2)	80 (16.6)	77 (11.6)	21 (23.1)	26 (27.4)	57 (23.0)	92 (22.1)	106 (27.0)	93 (22.5)	151 (17.0)	
Obesity class 2	309 (5.9)	15 (3.4)	18 (1.6)	18 (3.7)	13 (2.0)	11 (12.1)	9 (9.5)	23 (9.3)	45 (10.8)	58 (14.8)	49 (11.8)	50 (5.6)	
Obesity class 3	222 (4.2)	10 (2.3)	7 (0.6)	7 (1.4)	11 (1.7)	12 (13.2)	12 (12.6)	11 (4.4)	39 (9.4)	40 (10.2)	44 (10.6)	29 (3.3)	
**Demographic factors**													
Age, mean (SD), y	70.73 (11.51)	64.21 (12.68)	72.70 (11.99)	70.04 (11.50)	73.38 (11.16)	69.54 (11.01)	67.64 (10.14)	68.62 (11.59)	69.43 (11.08)	68.59 (10.25)	71.22 (10.77)	72.31 (10.06)	<.001
Age group, y													
40-59	899 (17.2)	173 (39.0)	161 (14.8)	95 (19.7)	72 (10.8)	19 (20.9)	19 (20.0)	43 (17.3)	90 (21.6)	72 (18.4)	64 (15.5)	91 (10.2)	<.001
60-79	3043 (58.2)	209 (47.1)	565 (51.8)	271 (56.1)	381 (57.2)	56 (61.5)	63 (66.3)	160 (64.5)	245 (58.8)	262 (66.8)	250 (60.4)	581 (65.4)	
≥80	1287 (24.6)	62 (14.0)	365 (33.5)	117 (24.2)	213 (32.0)	16 (17.6)	13 (13.7)	45 (18.1)	82 (19.7)	58 (14.8)	100 (24.2)	216 (24.3)	
Sex													
Female	2174 (41.6)	83 (18.7)	438 (40.1)	216 (44.7)	291 (43.7)	39 (42.9)	35 (36.8)	99 (39.9)	197 (47.2)	188 (48.0)	223 (53.9)	365 (41.1)	<.001
Male	3055 (58.4)	361 (81.3)	653 (59.9)	267 (55.3)	375 (56.3)	52 (57.1)	60 (63.2)	149 (60.1)	220 (52.8)	204 (52.0)	191 (46.1)	523 (58.9)	
Educational attainment													
Less than high school	301 (5.8)	12 (2.7)	64 (5.9)	59 (12.2)	19 (2.9)	7 (7.7)	11 (11.6)	16 (6.5)	40 (9.6)	26 (6.6)	29 (7.0)	18 (2.0)	<.001
High school diploma or GED	1113 (21.3)	12 (2.7)	131 (12.0)	110 (22.8)	142 (21.3)	44 (48.4)	30 (31.6)	65 (26.2)	194 (46.5)	152 (38.8)	156 (37.7)	77 (8.7)	
Some college	1294 (24.7)	11 (2.5)	170 (15.6)	153 (31.7)	170 (25.5)	26 (28.6)	42 (44.2)	81 (32.7)	128 (30.7)	134 (34.2)	160 (38.6)	219 (24.7)	
College degree or above	2521 (48.2)	409 (92.1)	726 (66.5)	161 (33.3)	335 (50.3)	14 (15.4)	12 (12.6)	86 (34.7)	55 (13.2)	80 (20.4)	69 (16.7)	574 (64.6)	
Annual income, $													
>150 000	1150 (22.2)	245 (55.2)	284 (26.0)	55 (11.4)	134 (20.1)	11 (12.1)	6 (6.3)	43 (17.3)	38 (9.1)	37 (9.4)	38 (9.2)	259 (29.2)	<.001
50 000-150 000	2292 (43.8)	143 (32.2)	457 (41.9)	195 (40.4)	317 (47.6)	40 (44.0)	49 (51.6)	117 (47.2)	180 (43.2)	182 (46.4)	194 (46.9)	418 (47.1)	
20 000-49 999	1227 (23.5)	27 (6.1)	199 (18.2)	155 (32.1)	152 (22.8)	30 (33.0)	28 (29.5)	62 (25.0)	145 (34.8)	130 (33.2)	132 (31.9)	167 (18.8)	
<20 000	560 (10.7)	29 (6.5)	151 (13.8)	78 (16.1)	63 (9.5)	10 (11.0)	12 (12.6)	26 (10.5)	54 (12.9)	43 (11.0)	50 (12.1)	44 (5.0)	
**Behavioral factors**													
Hours of sleep													
6-8	3927 (75.1)	382 (86.0)	850 (77.9)	334 (69.2)	494 (74.2)	62 (68.1)	63 (66.3)	188 (75.8)	270 (64.7)	272 (69.4)	282 (68.1)	730 (82.2)	<.001
<6	956 (18.3)	41 (9.2)	159 (14.6)	125 (25.9)	128 (19.2)	23 (25.3)	28 (29.5)	48 (19.4)	123 (29.5)	106 (27.0)	101 (24.4)	74 (8.3)	
>8	346 (6.6)	21 (4.7)	82 (7.5)	24 (5.0)	44 (6.6)	6 (6.6)	4 (4.2)	12 (4.8)	24 (5.8)	14 (3.6)	31 (7.5)	84 (9.5)	
Physical activity level^d^													
Underactive	4806 (91.9)	444 (100)	1030 (94.4)	439 (90.9)	622 (93.4)	82 (90.1)	78 (82.1)	214 (86.3)	362 (86.8)	334 (85.2)	364 (87.9)	837 (94.3)	<.001
Active	423 (8.1)	0	61 (5.6)	44 (9.1)	44 (6.6)	9 (9.9)	17 (17.9)	34 (13.7)	55 (13.2)	58 (14.8)	50 (12.1)	51 (5.7)	
REAP-S score, mean (SD)^e^	33.12 (6.24)	37.68 (5.99)	34.44 (6.01)	31.27 (5.41)	31.93 (5.66)	29.60 (6.30)	30.23 (6.14)	31.25 (6.01)	30.14 (5.52)	30.68 (5.40)	30.92 (5.58)	35.78 (5.70)	<.001

Abbreviations: BMI, body mass index (calculated as weight in kilograms divided by height in meters squared); GED, General Educational Development; PI, Pacific Islander; REAP-S, Rapid Eating Assessment for Participants–Shortened Version; WHO, World Health Organization.

^a^ Data are presented as the number (percentage) of participants unless otherwise indicated.

^b^ Includes less populous groups such as CHamorro/CHamoru, Fijian, Marshallese, Samoan, Tahitian, and Tongan.

^c^ Underweight was defined as a BMI less than 18.5; healthy weight, 18.5 to less than 25.0; overweight, 25.0 to less than 30.0; obesity class 1, 30.0 to less than 35.0; obesity class 2, 35.0 to less than 40.0; and obesity class 3, 40.0 or higher.

^d^ Underactive was defined as less than 60 minutes of strenuous or 150 minutes of moderate to vigorous activity per week and active as at least 60 minutes of strenuous or 150 minutes of moderate to vigorous activity per week.

^e^ Score ranges from 4 to 52, with higher scores indicating a healthier diet.

Obesity prevalence differed by race and ethnicity. In general, the prevalence of obesity was highest among multiracial individuals compared with single-race Asian groups and some of the Native Hawaiian and Other Pacific Islander groups (Table 1 and Figure). Among multiracial groups, people who identified as Asian, Pacific Islander, and White had the highest prevalence of obesity (204 of 392 [52.0%]), followed by people who identified as Pacific Islander and White (186 of 414 [44.9%]), Asian and Pacific Islander (176 of 417 [42.2%]), and Asian and White (91 of 248 [36.7%]). Among single-race groups, prevalence of obesity was highest among Other Pacific Islander (47 of 95 [49.5%]) and Native Hawaiian (44 of 91 [48.4%]) people. Among the Asian single-race groups, prevalence of obesity was highest among Filipino people (105 of 483 [21.7%]) and lowest among Chinese individuals (93 of 1091 [8.5%]). White individuals had an obesity prevalence of 25.9% (230 of 888). Differences compared with White people remained statistically significant after adjustment for multiple comparisons.

**Figure.**
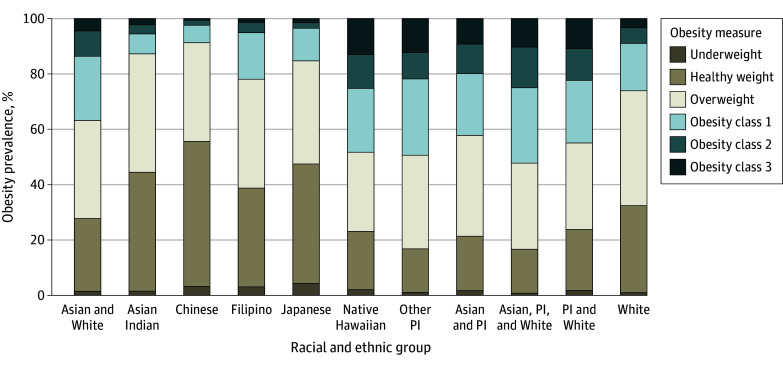
Weight Category Distribution Among 5229 Survey Participants in the Cardiovascular Disease Among Asians and Pacific Islanders Study Per World Health Organization standard body mass index (BMI; calculated as weight in kilograms divided by height in meters squared) cutoffs, underweight was less than 18.5; healthy weight, 18.5 to less than 25.0; overweight, 25.0 to less than 30; obesity class 1, 30.0 to less than 35.0; obesity class 2, 35.0 to less than 40.0; and obesity class 3, 40.0 or higher. Other Pacific Islander (PI) includes less populous groups such as CHamorro/CHamoru, Fijian, Marshallese, Samoan, Tahitian, and Tongan individuals.

There were distinct differences in the log odds of obesity by race and ethnicity (Table 2). In our unadjusted model, Asian Indian, Chinese, and Japanese individuals had lower odds of obesity compared with White individuals. In contrast, Native Hawaiian and Other Pacific Islander individuals and all of the multiracial groups had increased odds of obesity compared with White people.

**Table 2.** Multivariable Logistic Regression of the Odds of Obesity by Racial and Ethnic Group in the Cardiovascular Disease Among Asians and Pacific Islanders Study^a^

Racial and ethnic group	Model 1^b^		Model 2^c^		Model 3^d^		Model 4^e^	
	OR (95% CI)	*P* value	OR (95% CI)	*P* value	OR (95% CI)	*P* value	OR (95% CI)	*P* value
**Single race**									
Asian Indian	0.41 (0.30-0.56)	<.001	0.24 (0.17-0.34)	<.001	0.28 (0.19-0.39)	<.001	0.29 (0.20-0.40)	<.001
Chinese	0.27 (0.20-0.34)	<.001	0.25 (0.19-0.32)	<.001	0.25 (0.19-0.33)	<.001	0.22 (0.17-0.29)	<.001
Filipino	0.79 (0.61-1.03)	.09	0.69 (0.52-0.90)	.007	0.60 (0.45-0.80)	<.001	0.46 (0.35-0.62)	<.001
Japanese	0.51 (0.39-0.66)	<.001	0.51 (0.39-0.67)	<.001	0.49 (0.37-0.63)	<.001	0.38 (0.29-0.50)	<.001
Native Hawaiian only	2.68 (1.73-4.15)	<.001	2.47 (1.57-3.90)	<.001	2.02 (1.27-3.22)	.003	1.52 (0.94-2.45)	.09
Other Pacific Islander^f^	2.80 (1.82-4.31)	<.001	2.33 (1.49-3.63)	<.001	1.90 (1.20-3.00)	.006	1.46 (0.91-2.32)	.12
White	1 [Reference]	NA	1 [Reference]	NA	1 [Reference]	NA	1 [Reference]	NA
**Multiracial**									
Asian and Pacific Islander	2.09 (1.63-2.67)	<.001	1.89 (1.46-2.43)	<.001	1.52 (1.16-2.00)	.003	1.15 (0.86-1.52)	.34
Asian, Pacific Islander, and White	3.10 (2.42-3.98)	<.001	2.77 (2.14-3.58)	<.001	2.29 (1.75-3.00)	<001	1.80 (1.37-2.38)	<.001
Asian and White	1.66 (1.23-2.23)	<.001	1.40 (1.02-1.90)	.04	1.24 (0.90-1.69)	.18	1.00 (0.72-1.38)	.99
Pacific Islander and White	2.33 (1.83-2.98)	<.001	2.35 (1.83-3.03)	<.001	1.94 (1.49-2.54)	<001	1.55 (1.18-2.04)	.002

Abbreviations: NA, not applicable; OR, odds ratio.

^a^ Estimates based on multiple imputation by chained equations using 5 imputed datasets. Obesity was defined as having a body mass index of 30.0 or greater (defined as weight in kilograms divided by height in meters squared).

^b^ Adjusted for race and ethnicity only.

^c^ Adjusted for race and ethnicity plus demographic factors (age, sex).

^d^ Adjusted for race and ethnicity plus demographic and socioeconomic factors (educational level, annual income).

^e^ Adjusted for race and ethnicity plus demographic, socioeconomic, and behavioral factors (sleep, physical activity, and diet).

^f^ Includes less populous groups such as CHamorro/CHamoru, Fijian, Marshallese, Samoan, Tahitian, and Tongan.

These associations remained robust, but with slightly attenuated ORs, when accounting for age and sex (model 2). Unlike in model 1, Filipino individuals in model 2 had significantly lower odds of obesity compared with White individuals. Decreased odds of obesity remained for the other single-race Asian groups, while increased odds of obesity remained for Native Hawaiian and Pacific Islander individuals and the multiracial groups.

Educational attainment and income accounted for some of the differences in the odds of obesity by race and ethnicity (model 3). Trends remained similar for all groups except for multiracial Asian and White people, for whom there was no difference in odds of obesity compared with White people (OR, 1.24; 95% CI, 0.90-1.69; *P* = .18).

Finally, after additional adjustment for sleep duration, physical activity, and diet (model 4), all single-race Asian groups had significantly lower odds of obesity compared with White people: Asian Indian (OR, 0.29; 95% CI, 0.20-0.40), Chinese (OR, 0.22; 95% CI, 0.17-0.29), Filipino (OR, 0.46; 95% CI, 0.35-0.62), and Japanese (OR, 0.38, 95% CI, 0.29-0.50). There was no difference in odds of obesity between Native Hawaiian (OR, 1.52; 95% CI, 0.94-2.45) or Other Pacific Islander (OR, 1.46; 95% CI, 0.91-2.32) individuals and White people. Finally, in model 4, only multiracial individuals who were Asian, Pacific Islander, and White (OR, 1.80; 95% CI, 1.37-2.38) or Pacific Islander and White (OR, 1.55; 95% CI, 1.18-2.04) had significantly higher odds of obesity compared with White people.

In supplemental analyses of 540 629 adults aged 40 years or older with complete race data, there were some slight differences in the prevalence of obesity for the full EHR data compared with the EHR-survey linked data (eTable 1 and eFigure 3 in Supplement 1). Individuals who identified as Other Pacific Islander had the highest prevalence of obesity (3308 of 5516 [60.0%]), followed by Asian, Pacific Islander, and White people (3015 of 5748 [52.5%]); Native Hawaiian people (995 of 2020 [49.3%]); and Pacific Islander and White people (2758 of 5700 [48.4%]). These 4 groups had the 4 highest obesity prevalence rates in both EHR and survey-linked data. When examining the unadjusted and age- and sex-adjusted associations between race and ethnicity and obesity using EHR data only (eTable 2 in Supplement 1), the results were the same as in the main analysis.

We further examined whether these associations changed when Asian-specific obesity cutoffs (BMI≥27.5) were applied to Asian Indian, Chinese, Filipino, and Japanese individuals (eTable 3 in Supplement 1) and both EHR and survey-linked data were used. In our demographic and socioeconomic adjusted model (model 3), Asian Indian and Chinese individuals continued to have lower odds of obesity while Filipino and Japanese individuals had higher odds of obesity compared with White people. There was no difference in odds of obesity between Filipino or Japanese individuals and White individuals when accounting for health behaviors (model 4).

Trends were similar when applying Asian obesity cutoffs to single-race Asian groups in the EHR data. The prevalence of obesity among single-race Asian people increased when Asian-specific cutoffs were applied; Filipinos had the highest prevalence (16 799 of 41 440 [40.5%]) (eTable 4 in Supplement 1). In age- and sex-adjusted models (eTable 5 in Supplement 1), only Chinese individuals had lower odds of obesity compared with White people, while Asian Indian, Filipino, and Japanese individuals had increased odds of obesity.

## Discussion

Examination of disparities in obesity by race and ethnicity often do not consider disaggregated Asian and Pacific Islander subgroups or to multiracial groups. This is particularly troublesome given the steady increase in Asian and Pacific Islander people and people who identify as multiracial in the US. Similar to recent studies,^7,8^ our study found that single-race Asian groups had lower odds of obesity compared with White individuals when using standard BMI cutoffs. However, when applying Asian-specific BMI cutoffs,^7^ the prevalence of obesity among single-race Asian groups was similar to that among single-race White individuals. Moreover, we found that obesity prevalence was higher among Pacific Islander groups compared with Asian groups and White individuals.^32,33^

Our study provides new insights into the burden of obesity among multiracial groups. In both EHR and survey-linked data, the prevalence of obesity was higher for multiracial groups than for both single-race Asian and White people when using standard BMI cutoffs. Although demographic, socioeconomic, and health behavior factors explained some of the differences in multiracial Asian and Pacific Islander groups compared with White people, increased odds of obesity were still seen among multiracial people who identified as Asian, Pacific Islander, and White or Pacific Islander and White.

These results emphasize the importance of disaggregating data on Asian and Pacific Islander people when studying health and clinical data. Previous studies using state and national data also found a heterogeneous prevalence of obesity among Asian subgroups specifically.^7,8,16,17,22,31^

Furthermore, accounting for demographic, socioeconomic, and health behavior factors did not change the comparative prevalence of obesity across racial and ethnic groups except for single-race Native Hawaiian and Pacific Islander individuals and the multiracial Asian and White and Asian and Pacific Islander groups. These results suggest that there may be additional factors, such as experiences of stress,^34^ immigrant health selectivity,^35^ and acculturation,^36^ that could contribute to differences in prevalence of obesity among these groups.

To our knowledge, this study provides one of the first examinations into obesity disparities among multiracial Asian and Pacific Islander populations. Previous studies have varied in how they categorized multiracial individuals, classifying them in an “other” category (eg, other Asian),^8^ including them with the largest single-race group included (eg, Asian),^22^ or excluding them entirely because of small sample sizes.^7,8^ Our study provides an initial look into the burden of obesity among multiracial individuals by providing distinct groups with large enough sample sizes to detect possible differences.

### Strengths and Limitations

Our study has a number of strengths. First, we provide a comprehensive examination of disaggregated Asian and Pacific Islander groups. National survey data, such as the National Health and Nutrition Examination Survey, National Health Interview Survey, or Behavioral Risk Factor Surveillance System, may not provide comprehensive data about Asian and Pacific Islander groups with adequate sample sizes.^37,38^ Second, outcome data in the EHR are not subject to the same reporting biases as survey data. Third, our study augments EHR data by providing linked survey and health behavior data. The use of these data provides a more comprehensive view of obesity among EHR participants. In addition, our study provides an extensive look at the burden of obesity among multiracial groups.

Our results should be examined in light of some limitations. First, although we had large sample sizes of disaggregated Asian and Pacific Islander groups, we were unable to include other smaller groups (eg, Hmong, Thai, Vietnamese). Thus, our sample may not be generalizable to the larger national population, although we found trends similar to those in national studies.^7,8^ The population in our study was derived from members of 2 health care systems in Northern California and Hawai’i and therefore may not represent the national Asian and Pacific Islander population. Additionally, our survey population was selected by case-control matching on cardiovascular disease status with oversampling of Asian and Pacific Islander racial and ethnic groups. Although our results are similar to those of previous studies,^7,8,31,32,33^ it is possible that this sample may have a different health and sociodemographic profile compared with the larger Asian and Pacific Islander population. We were also only able to disaggregate Native Hawaiian individuals from the larger Pacific Islander group. While we provided expansive categories for multiracial individuals, we were unable to further explore smaller groups (eg, Filipino and White, Japanese and Native Hawaiian). Future work should examine more detailed multiracial groups. We also did not have complete socioeconomic and behavioral data for the survey sample. Although imputation is a feasible solution, health care organizations should work to expand their collection of patient-reported data, as studies like ours show the critical need for such information to understand differences in patient outcomes in large samples. In addition, although our study highlights disparities in obesity, BMI is an imperfect measure of cardiometabolic health, especially among certain groups like athletes and older adults.^39,40,41^

## Conclusions

This study found that multiracial Asian and Pacific Islander individuals had an increased prevalence of obesity compared with many of their single-race counterparts. As public health and clinical practice address the increasing rates of obesity in the US, it is important to acknowledge demographic changes within the US population. The growing population of individuals who identify as multiracial will present new groups to consider in the future. In addition, the lack of standardized methods to understand this population could lead to multiracial people’s invisibility from discussions of health equity. Clinical care must also consider the unique needs that multiracial individuals may have.

## Notes

Supplement 1.
eMethods.
**eFigure 1.** Example Case-Control Response Count Among the Asian and Pacific Islander Racial and Ethnic Group **eFigure 2.** Comparison of Cases and Controls by Sex **eTable 1.** Participant Characteristics Among All EHR Participants in the CASPER Study **eTable 2.** Multivariable Regression of the Odds of Obesity by Race and Ethnicity Group Among EHR Participants in the CASPER Study **eTable 3.** Multivariable Regression of the Odds of Obesity by Race and Ethnicity in the CASPER Study Using Asian-Specific Cutoffs **eTable 4.** Prevalence of Obesity by BMI Cutoff in the CASPER Study **eTable 5.** Multivariable Regression of the Odds of Obesity by Race and Ethnicity Among CASPER EHR Participants Using Asian-Specific Cutoffs **eFigure 3.** Weight Category Distribution Using WHO and Asian-Specific Cutoffs Among EHR Participants in the CASPER Study

Supplement 2.
Data Sharing Statement
