# Supplementary material for: Obesity Disparities Among Adult Single-Race and Multiracial Asian and Pacific Islander Populations
Source: JAMA Netw Open. 2024 Mar 19;7(3):e240734. doi: 10.1001/jamanetworkopen.2024.0734 (PMC10951735; doi:10.1001/jamanetworkopen.2024.0734)
Supplement: Supplement 1. — eMethods. eFigure 1. Example Case-Control Response Count Among the Asian and Pacific Islander Racial and Ethnic Group eFigure 2. Comparison of Cases and Controls by Sex eTable 1. Participant Characteristics Among All EHR Participants in the CASPER Study eTable 2. Multivariable Regression of the Odds of Obesity by Race and Ethnicity Group Among EHR Participants in the CASPER Study eTable 3. Multivariable Regression of the Odds of Obesity by Race and Ethnicity in the CASPER Study Using Asian-Specific Cutoffs eTable 4. Prevalence of Obesity by BMI Cutoff in the CASPER Study eTable 5. Multivariable Regression of the Odds of Obesity by Race and Ethnicity Among CASPER EHR Participants Using Asian-Specific Cutoffs eFigure 3. Weight Category Distribution Using WHO and Asian-Specific Cutoffs Among EHR Participants in the CASPER Study [file jamanetwopen-e240734-s001.pdf]

Supplementary Online Content

Bacong AM, Gibbs S, Rosales AG, et al. Obesity disparities among adult single-race and multiracial Asian and Pacific Islander populations. *JAMA Netw Open*. 2024;7(3):e240734. doi:10.1001/jamanetworkopen.2024.0734

eMethods

eFigure 1. Example Case-Control Response Count Among the Asian and Pacific Islander Racial and Ethnic Group

eFigure 2. Comparison of Cases and Controls by Sex

eTable 1. Participant Characteristics Among All EHR Participants in the CASPER Study

eTable 2. Multivariable Regression of the Odds of Obesity by Race and Ethnicity Group Among EHR Participants in the CASPER Study

eTable 3. Multivariable Regression of the Odds of Obesity by Race and Ethnicity in the CASPER Study Using Asian-Specific Cutoffs

eTable 4. Prevalence of Obesity by BMI Cutoff in the CASPER Study

eTable 5. Multivariable Regression of the Odds of Obesity by Race and Ethnicity Among CASPER EHR Participants Using Asian-Specific Cutoffs

eFigure 3. Weight Category Distribution Using WHO and Asian-Specific Cutoffs Among EHR Participants in the CASPER Study

This supplementary material has been provided by the authors to give readers additional information about their work.

© 2024 Bacong AM et al. *JAMA Network Open*.

eMethods. CASPER Survey Sampling Strategy

The primary aims of the Cardiovascular Disease Among Asians and Pacific Islanders (CAPSER) Study were to examine the burden of cardiovascular disease and related contributors for single and multiple race Asian and Pacific Islander people as compared to the Non-Hispanic White population. Originally, the survey sampling plan was to focus on 10 racial and ethnic groups (seven single race and four multiracial), with the goal of obtaining an equal number of case and control respondents within each group. The six single race groups included “Asian Indian”, “Chinese”, “Filipino”, “Japanese”, “Native Hawaiian Only”, and “Non-Hispanic White”. The four multiracial groups included “Asian & White”, “Pacific Islander & Asian”, “Pacific Islander, Asian, & White”, and “Pacific Islander & White”. Although the CASPER Study considered other multiple race groups (e.g., “Asian and Black”, “Pacific Islander & Black”, “Asian and Hispanic”), the study team chose to forgo sampling of these groups due to these groups’ lack of representation at the two catchment areas. Among those sampled, cases were defined as individuals with any prevalent diagnosed cardiovascular disease (CVD) (e.g., coronary heart disease/myocardial infarction, peripheral vascular disease, and stroke) while controls were defined as individuals without recorded cardiovascular disease at the time of the survey.

The plan was to stagger survey implementation to collect data from approximately 50% of our cases first. Based on the variable frequency distributions of a few key variables (age, gender, geographic region) from our respondents with events, we planned to select an equal size frequency-matched sample of patients without CVD events from each group. The total planned survey respondent population was to include approximately 10,000 people (1/2 cases and 1/2 controls), and we estimated a 60% response rate, based on previous experience.

Our estimates of sample sizes for CVD cases in each of the racial and ethnic groups from preliminary data included in the proposal varied from under 200 to over 10,000, due to differences in population sizes and CVD rates. While we recognized that from a statistical standpoint, it would be ideal to have an equal number of respondents from each of the racial and ethnic groups (with an equal number of cases and controls within each group), we also understood that this would be challenging to achieve. There were differences in the total size of available sample for each racial and ethnic group, differences in the proportions of cases and controls within each group, and differences in response rates at the 2 sites, complicated by differences in the proportion of individual racial and ethnic groups represented at each site. We also wanted to obtain about the same number of total respondents from each site.

There were also a number of other refinements to study methods that took place prior to survey implementation:

- We redefined the cohort to include a larger time period
- We found we had enough cases to add an 11th racial and ethnic group – “Other Pacific Islanders”
- We also wanted to ensure that we would have sufficient representation of CVD subtypes (CHD, Stroke and PVD) among our case respondents
- Time constraints prohibited us from waiting for recruitment of all cases to be completed before matching, and recruitment of controls, necessitating a continuous iterative recruitment process

In consultation with the study biostatistician, our strategy was to attempt to obtain as close to an equal number of case respondents[1] as possible among each of the 11 groups (approximately 227 each, from each site), stratified by study site (KPHI and PAMF) and CVD subtype (CHD, Stroke and PVD), to yield a total number of 5,000 case respondents. To start, 227 cases were selected from racial and ethnic groups that had this many available cases or more at each site. For those groups that did not have 227 cases, all cases were selected. The remaining cases to fill were selected from those groups that had more than 227 cases in an iterative process. Each step would assess how many cases needed to be filled from each race/ethnicity group and whether each remaining race/ethnicity group had sufficient sample left to fill the need. If a race/ethnicity group did not have sufficient sample, then each remaining race/ethnicity group would fill up to the level of the race/ethnicity group with the lowest N remaining. Then the process would repeat until the sample selected reached 2,500 cases. Below is an example:

For instance: There are three race/ethnicity groups with more than 227 cases – Japanese -1,000, Pacific Islander & Asian – 1,000, and Chinese 300, leaving 1,250 cases remaining to be selected (1,250 already selected into

---

© 2024 Bacong AM et al. *JAMA Network Open*.

other groups). First, select 73 cases from each of three groups to get to 300 for each race/ethnicity group. 1,104 cases remain which are split between Japanese (552) and Asian and Pacific Islanders (552).

The process for matching controls followed the method mentioned above and described in the proposal below – frequency matching controls to case responders based on key variables, age, gender, and geographic region (study site). We began mailing case waves first so that we could observe the overall case response rate and get counts of responses by race/ethnicity, age, gender and study site - for instance the number of survey responders that were Japanese Age 70-79 Males at KP-Hawaii. By April 2018 we had mailed surveys to 2,500 cases across 4 waves and had received over 1,100 responses.

Simultaneously from our pool of controls we generated a frequency distribution of controls stratified by race/ethnicity, age, gender, and study site. We applied the study site specific survey response rate (~46% at Kaiser Permanente-Hawaii) at that time in cases to the number of case responders in a race/ethnicity, age, gender, and study site group to generate the number of controls to select to each group. While this yielded approximately 2,500 controls across 110 race/ethnicity, age, and gender groups at each site we planned to start with 3 waves totaling approximately 1,750 controls. The groups with the smallest counts were prioritized to be sent in the first 3 waves because of the higher variability of these groups potentially necessitating adjustments to achieve the same number of responses as the cases for that group. **eFigure 1** below is a screenshot of the spreadsheet and several groups showing the cases (selected and responded) and control selections.

© 2024 Bacong AM et al. *JAMA Network Open*.

eFigure 1. Example Case-Control Response Count Among the Asian and Pacific Islander Racial and Ethnic Group

Obs	Race/Ethnicity Group	Age Category	Gender	Cases Selected Count	Cases Responded Count	Controls Pool Count	Controls to Select Count	Controls Selected Count
102	Asian + Pacific Islander	50-59	M	28	13	584	28	28
103	Asian + Pacific Islander	60-69	M	45	17	403	37	37
104	Asian + Pacific Islander	70-79	M	63	25	190	54	54
105	Asian + Pacific Islander	80+	M	28	17	63	37	37
				2500	1159	60702	2493	2482
KP-Hawaii Response Rate 1159/2500=46.36%								

Finally, in July 2018 we compared the case and control responses at each study site by race/ethnicity, age and gender respectively. We observed that our control responses skewed older and female so we then adjusted the remaining control wave to be younger and more male. **eFigure 2** below shows the gender 2-by-2 table with survey responses in July 2018.

© 2024 Bacong AM et al. *JAMA Network Open*.

eFigure 2. Comparison of Cases and Controls by Sex

CASPER Response Distributions by Sex and Case/Control
Actual

The FREQ Procedure

Frequency<br>Percent<br>Row Pct<br>Col Pct

Table of sex by type			
	type		
sex(Gender)	Control	Case	Total
**F**	330<br>15.94<br>34.13<br>49.62	637<br>30.77<br>65.87<br>45.34	967<br>46.71
**M**	335<br>16.18<br>30.37<br>50.38	768<br>37.10<br>69.63<br>54.66	1103<br>53.29
**Total**	665<br>32.13	1405<br>67.87	2070<br>100.00

© 2024 Bacong AM et al. *JAMA Network Open*.

eTable 1. Participant Characteristics Among All Electronic Health Record Participants in the Cardiovascular Disease Among Asians and Pacific Islanders (CASPER) Study, N = 540,629

Race Group	Total	Asian Indian	Chinese	Filipino	Japanese	Native Hawaiian Only	Other Pacific Islander	Asian & White	Pacific Islander & Asian	Pacific Islander & Asian & White	Pacific Islander & White	Non-Hispanic White	p-value
**N**	**540,629**	**42,599**	**50,544**	**41,440**	**27,117**	**2,020**	**5,516**	**6,312**	**8,492**	**5,748**	**5,700**	**345,141**	
**Variable**	**n (%) or Mean (SD)**	**n (%) or Mean (SD)**	**n (%) or Mean (SD)**	**n (%) or Mean (SD)**	**n (%) or Mean (SD)**	**n (%) or Mean (SD)**	**n (%) or Mean (SD)**	**n (%) or Mean (SD)**	**n (%) or Mean (SD)**	**n (%) or Mean (SD)**	**n (%) or Mean (SD)**	**n (%) or Mean (SD)**	
**Clinical Outcomes**													
BMI	27.12 (5.97)	25.99 (4.42)	23.94 (4.14)	26.97 (5.44)	25.29 (5.18)	30.89 (7.51)	32.41 (7.28)	28.17 (5.91)	30.01 (7.56)	31.09 (7.26)	30.77 (7.54)	27.56 (6.07)	<0.001
Obese (≥30)	135115 (25.0)	7014 (16.5)	3447 (6.8)	10114 (24.4)	4465 (16.5)	995 (49.3)	3308 (60.0)	2076 (32.9)	3826 (45.1)	3015 (52.5)	2758 (48.4)	94097 (27.3)	<0.001
**BMI Categories**													
Standard Weight Category													<0.001
Underweight	16152 (3.0)	1177 (2.8)	2840 (5.6)	1083 (2.6)	1676 (6.2)	24 (1.2)	38 (0.7)	92 (1.5)	283 (3.3)	75 (1.3)	95 (1.7)	8769 (2.5)	
Healthy/Normal Weight	195392 (36.1)	17427 (40.9)	29798 (59.0)	15025 (36.3)	12377 (45.6)	412 (20.4)	698 (12.7)	1906 (30.2)	1795 (21.1)	1059 (18.4)	1115 (19.6)	113780 (33.0)	
Overweight	193970 (35.9)	16981 (39.9)	14459 (28.6)	15218 (36.7)	8599 (31.7)	589 (29.2)	1472 (26.7)	2238 (35.5)	2588 (30.5)	1599 (27.8)	1732 (30.4)	128495 (37.2)	
Obese Class 1	88849 (16.4)	5327 (12.5)	2629 (5.2)	6897 (16.6)	3211 (11.8)	473 (23.4)	1594 (28.9)	1257 (19.9)	1963 (23.1)	1565 (27.2)	1419 (24.9)	62514 (18.1)	
Obese Class 2	29421 (5.4)	1291 (3.0)	572 (1.1)	2295 (5.5)	937 (3.5)	275 (13.6)	908 (16.5)	570 (9.0)	1031 (12.1)	809 (14.1)	707 (12.4)	20026 (5.8)	

© 2024 Bacong AM et al. *JAMA Network Open*.

Race Group	Total	Asian Indian	Chinese	Filipino	Japanese	Native Hawaiian Only	Other Pacific Islander	Asian & White	Pacific Islander & Asian	Pacific Islander & Asian & White	Pacific Islander & White	Non-Hispanic White	p-value
**N**	**540,629**	**42,599**	**50,544**	**41,440**	**27,117**	**2,020**	**5,516**	**6,312**	**8,492**	**5,748**	**5,700**	**345,141**	
**Variable**	**n (%) or Mean (SD)**	**n (%) or Mean (SD)**	**n (%) or Mean (SD)**	**n (%) or Mean (SD)**	**n (%) or Mean (SD)**	**n (%) or Mean (SD)**	**n (%) or Mean (SD)**	**n (%) or Mean (SD)**	**n (%) or Mean (SD)**	**n (%) or Mean (SD)**	**n (%) or Mean (SD)**	**n (%) or Mean (SD)**	
Obese Class 3 (40+)	16845 (3.1)	396 (0.9)	246 (0.5)	922 (2.2)	317 (1.2)	247 (12.2)	806 (14.6)	249 (3.9)	832 (9.8)	641 (11.2)	632 (11.1)	11557 (3.3)	
**Demographic Factors**													
Mean Age (SD)	59.14 (13.37)	49.63 (9.96)	63.90 (15.17)	56.78 (13.16)	57.25 (12.32)	57.77 (12.57)	54.49 (11.12)	54.26 (11.32)	58.16 (12.70)	56.72 (12.29)	59.35 (13.28)	60.74 (13.19)	<0.001
Age Group													<0.001
40-59 Years Old	301487 (55.8)	36569 (85.8)	32933 (65.2)	25235 (60.9)	11906 (43.9)	1244 (61.6)	3898 (70.7)	4476 (70.9)	4940 (58.2)	3609 (62.8)	3198 (56.1)	173479 (50.3)	
60-79 Years Old	188155 (34.8)	5163 (12.1)	13522 (26.8)	13804 (33.3)	10009 (36.9)	635 (31.4)	1423 (25.8)	1627 (25.8)	2976 (35.0)	1854 (32.3)	1991 (34.9)	135151 (39.2)	
80+ Years Old	50987 (9.4)	867 (2.0)	4089 (8.1)	2401 (5.8)	5202 (19.2)	141 (7.0)	195 (3.5)	209 (3.3)	576 (6.8)	285 (5.0)	511 (9.0)	36511 (10.6)	
Sex													
Female	286779 (53.0)	18924 (44.4)	28468 (56.3)	23299 (56.2)	15450 (57.0)	974 (48.2)	2817 (51.1)	3388 (53.7)	4169 (49.1)	3182 (55.4)	3090 (54.2)	183018 (53.0)	
Male	253850 (47.0)	23675 (55.6)	22076 (43.7)	18141 (43.8)	11667 (43.0)	1046 (51.8)	2699 (48.9)	2924 (46.3)	4323 (50.9)	2566 (44.6)	2610 (45.8)	162123 (47.0)	<0.001

Note: BMI = Body Mass Index; Obesity was defined as having a BMI of 30 kg/m^2 or greater. Underweight was defined as having a BMI of 18.5 or lower. Healthy/Normal Weight was defined as a BMI between 18.5 to 24.9. Overweight was defined as having a BMI between 25.0 to 29.9. Obese Class 1 was defined as a BMI between 30.0 to 34.9. Obese Class 2 was defined as a BMI between 35.0 and 39.9. Obese Class 3 was defined as a BMI greater than or equal to 40.0. “Other Pacific Islander” includes less populous groups such as CHamorro/CHamoru, Fijian, Marshallese, Samoan, Tongan, and Tahitian.

© 2024 Bacong AM et al. *JAMA Network Open*.

eTable 2. Multivariable Regression of the Odds of Obesity by Race and Ethnicity Group Among Electronic Health Record Participants in the Cardiovascular Disease Among Asians and Pacific Islanders (CASPER) Study, N = 540,629

	Model 1: Crude Model		Model 2: Age and Sex Adjusted	
**VARIABLES**	**OR (95% CI)**	**P-Value**	**OR (95% CI)**	**P-Value**
**Race and Ethnic Group**				
Non-Hispanic White (Ref.)	Ref.		Ref.	
Asian Indian	0.53 (0.51-0.54)	< .001	0.48 (0.47- 0.50)	< .001
Chinese	0.20 (0.19-0.20)	< .001	0.19 (0.18-0.20)	< .001
Filipino	0.86 (0.84-0.88)	< .001	0.85 (0.83-0.87)	< .001
Japanese	0.53 (0.51-0.54)	< .001	0.54 (0.52-0.56)	< .001
Native Hawaiian Only	2.59 (2.37-2.83)	< .001	2.53 (2.32-2.76)	< .001
Other Pacific Islander (single ethnic group)	4.00 (3.79-4.22)	< .001	3.85 (3.65-4.07)	< .001
Asian + White	1.31 (1.24-1.38)	< .001	1.26 (1.19-1.33)	< .001
Pacific Islander + Asian	2.19 (2.09-2.28)	< .001	2.14 (2.05-2.24)	< .001
Pacific Islander + Asian + White	2.94 (2.79-3.10)	< .001	2.89 (2.75-3.05)	< .001
Pacific Islander + White	2.50 (2.37-2.64)	< .001	2.49 (2.36-2.63)	< .001

Note. Estimates based on Multiple Imputation by Chained Equations (MICE) using 5 imputed datasets. Obesity was defined as having a body mass index (BMI) ≥ 30.0 kg/m^2. “Other Pacific Islander” includes less populous groups such as CHamorro/CHamoru, Fijian, Marshallese, Samoan, Tongan, and Tahitian.

© 2024 Bacong AM et al. *JAMA Network Open*.

eTable 3. Multivariable Regression of the Odds of Obesity by Race and Ethnicity in the Cardiovascular Disease Among Asians and Pacific Islanders (CASPER) Study Survey Participants Using Asian-Specific Cutoffs, N = 5,229

	Model 1: Race Only		Model 2: Race + Demographic Factors		Model 3: Race + Demographic + Socioeconomic Factors		Model 4: Race + Demographic + Socioeconomic + Behavioral	
**VARIABLES**	**OR (95% CI)**	**P-Value**	**OR (95% CI)**	**P-Value**	**OR (95% CI)**	**P-Value**	**OR (95% CI)**	**P-Value**
**Race and Ethnic Group**								
Non-Hispanic White (Ref.)	Ref.		Ref.		Ref.		Ref.	
Asian Indian	1.04 (0.80-1.34)	0.79	0.66 (0.50-0.87)	0.003	0.73 (0.55-0.96)	0.03	0.77 (0.58-1.02)	0.07
Chinese	0.74 (0.60-0.92)	0.006	0.73 (0.59-0.90)	0.0047	0.74 (0.59-0.92)	0.006	0.66 (0.53-0.83)	< .001
Filipino	1.76 (1.39-2.23)	< .001	1.64 (1.28-2.09)	< .001	1.49 (1.15-1.91)	0.002	1.18 (0.91-1.52)	0.22
Japanese	1.25 (1.00-1.56)	0.05	1.31 (1.05-1.65)	0.02	1.26 (1.00-1.58)	0.05	1.02 (0.80-1.29)	0.89
Native Hawaiian Only	2.68 (1.73-4.15)	< .001	2.49 (1.58-3.92)	< .001	2.13 (1.34-3.37)	0.001	1.61 (1.00-2.58)	0.05
Other Pacific Islander (single ethnic group)	2.80 (1.82-4.31)	< .001	2.36 (1.52-3.67)	< .001	2.01 (1.28-3.15)	0.003	1.56 (0.98-2.48)	0.06
Asian + White	1.66 (1.23-2.23)	< .001	1.42 (1.04-1.93)	0.03	1.29 (0.94-1.76)	0.11	1.05 (0.76-1.45)	0.74
Pacific Islander + Asian	2.09 (1.63-2.67)	< .001	1.92 (1.49-2.48)	< .001	1.62 (1.24-2.12)	< .001	1.24 (0.94-1.64)	0.12
Pacific Islander + Asian + White	3.10 (2.42-3.98)	< .001	2.82 (2.19-3.65)	< .001	2.43 (1.86-3.17)	< .001	1.94 (1.48-2.55)	< .001

© 2024 Bacong AM et al. *JAMA Network Open*.

Pacific Islander + White	2.33 (1.83-2.98)	< .001	2.39 (1.86-3.08)	< .001	2.05 (1.58-2.67)	< .001	1.65 (1.26-2.17)	< .001

Note. Demographic factors include age and sex. Socioeconomic factors include education and annual income. Health behavior factors include sleep, physical activity, and diet. Estimates based on Multiple Imputation by Chained Equations (MICE) using 5 imputed datasets. "Asian Obesity Cutoff" was defined as having a body mass index (BMI) ≥ 27.5 kg/m^2 and was applied for Asian Indian, Chinese, Filipino, and Japanese individuals only. Standard obesity cutoffs were defined as having a BMI ≥ 30.0 kg/m^2 and was applied to Native Hawaiian, Other Pacific Islander, and all multiracial individuals. "Other Pacific Islander" includes populous groups such as CHamorro/CHamoru, Fijian, Marshallese, Samoan, Tongan, and Tahitian.

© 2024 Bacong AM et al. *JAMA Network Open*.

eTable 4. Prevalence of Obesity by BMI Cutoff in the Cardiovascular Disease Among Asians and Pacific Islanders (CASPER) Study

Panel A: Survey Data (n=5,229)

Race Group	Total	Asian Indian	Chinese	Filipino	Japanese	Native Hawaiian Only	Other Pacific Islander	Asian & White	Pacific Islander & Asian	Pacific Islander & Asian & White	Pacific Islander & White	Non-Hispanic White
**N**	5,229	444	1,091	483	666	91	95	248	417	392	414	888
**Variable**	**n (%)**	**n (%)**	**n (%)**	**n (%)**	**n (%)**	**n (%)**	**n (%)**	**n (%)**	**n (%)**	**n (%)**	**n (%)**	**n (%)**
Asian BMI Cutoffs [a]	1707 (32.6)	118 (26.6)	225 (20.6)	184 (38.1)	202 (30.3)	44 (48.4)	47 (49.5)	91 (36.7)	176 (42.2)	204 (52.0)	186 (44.9)	230 (25.9)
WHO standard Cutoffs	1333 (25.5)	56 (12.6)	93 (8.5)	105 (21.7)	101 (15.2)	44 (48.4)	47 (49.5)	91 (36.7)	176 (42.2)	204 (52.0)	186 (44.9)	230 (25.9)

Panel B: Electronic Health Record Data (n=540,629)

Race Group	Total	Asian Indian	Chinese	Filipino	Japanese	Native Hawaiian Only	Other Pacific Islander	Asian & White	Pacific Islander & Asian	Pacific Islander & Asian & White	Pacific Islander & White	Non-Hispanic White
**N**	540,629	42,599	50,544	41,440	27,117	2,020	5,516	6,312	8,492	5,748	5,700	345,141
**Variable**	**n (%)**	**n (%)**	**n (%)**	**n (%)**	**n (%)**	**n (%)**	**n (%)**	**n (%)**	**n (%)**	**n (%)**	**n (%)**	**n (%)**
Asian BMI Cutoffs [a]	155535 (28.8)	12579 (29.5)	8488 (16.8)	16799 (40.5)	7594 (28.0)	995 (49.3)	3308 (60.0)	2076 (32.9)	3826 (45.1)	3015 (52.5)	2758 (48.4)	94097 (27.3)
WHO standard Cutoffs	135115 (25.0)	7014 (16.5)	3447 (6.8)	10114 (24.4)	4465 (16.5)	995 (49.3)	3308 (60.0)	2076 (32.9)	3826 (45.1)	3015 (52.5)	2758 (48.4)	94097 (27.3)

Note. [a] Asian-specific BMI cutoffs are 27.5 kg/m$^{2;}$ these cutoffs were applied to Asian Indian, Chinese, Filipino, and Japanese people only. WHO Obesity Standard was defined as having a BMI above 30 kg/m^2 and was applied to all groups. Estimates based on Multiple Imputation of Chained Equations (MICE) using 5 datasets. "Other Pacific Islander" includes less populous groups such as CHamorro/CHamoru, Fijian, Marshallese, Samoan, Tongan, and Tahitian..

© 2024 Bacong AM et al. *JAMA Network Open*.

eTable 5. Multivariable Regression of the Odds of Obesity by Race and Ethnicity Among Cardiovascular Disease Among Asians and Pacific Islanders (CASPER) EHR Participants Using Asian-Specific Cutoffs, N = 540,629

	Model 1: Race Only		Model 2: Race + Demographic Factors	
**VARIABLES**	**OR (95% CI)**	**P-Value**	**OR (95% CI)**	**P-Value**
**Race and Ethnic Group**				
Non-Hispanic White (Ref.)	Ref.		Ref.	
Asian Indian	1.12 (1.09-1.14)	< .001	1.03 (1.01-1.05)	.009
Chinese	0.54 (0.53-0.55)	< .001	0.53 (0.52-0.54)	< .001
Filipino	1.82 (1.78-1.86)	< .001	1.80 (1.76-1.84)	< .001
Japanese	1.04 (1.01-1.07)	.008	1.07 (1.04-1.10)	< .001
Native Hawaiian Only	2.59 (2.37-2.83)	< .001	2.53 (2.32-2.76)	< .001
Other Pacific Islander (single ethnic group)	4.00 (3.79-4.22)	< .001	3.86 (3.65-4.07)	< .001
Asian + White	1.31 (1.24-1.38)	< .001	1.26 (1.20-1.33)	< .001
Pacific Islander + Asian	2.19 (2.09-2.28)	< .001	2.14 (2.05-2.24)	< .001
Pacific Islander + Asian + White	2.94 (2.79-3.10)	< .001	2.90 (2.75-3.06)	< .001
Pacific Islander + White	2.50 (2.37-2.64)	< .001	2.49 (2.37-2.63)	< .001

Note. Models account for demographic, behavioral, and clinical factors. Estimates based on Multiple Imputation by Chained Equations (MICE) using 5 imputed datasets. "Asian Obesity Cutoff" was defined as having a body mass index (BMI) ≥ 27.5 kg/m^2 and was applied for Asian Indian, Chinese, Filipino, and Japanese individuals only. Standard obesity cutoffs were defined as having a BMI ≥ 30.0 kg/m^2 and was applied to Native Hawaiian, Other Pacific Islander, and all multiracial individuals. "Other Pacific Islander" includes populous groups such as CHamorro/CHamoru, Fijian, Marshallese, Samoan, Tongan, and Tahitian.

© 2024 Bacong AM et al. *JAMA Network Open*.

eFigure 3. Weight Category Distribution (WHO and Asian-Specific Cutoffs) Among Electronic Health Record Participants in the Cardiovascular Disease Among Asians and Pacific Islanders (CASPER) Study, N = 540,629

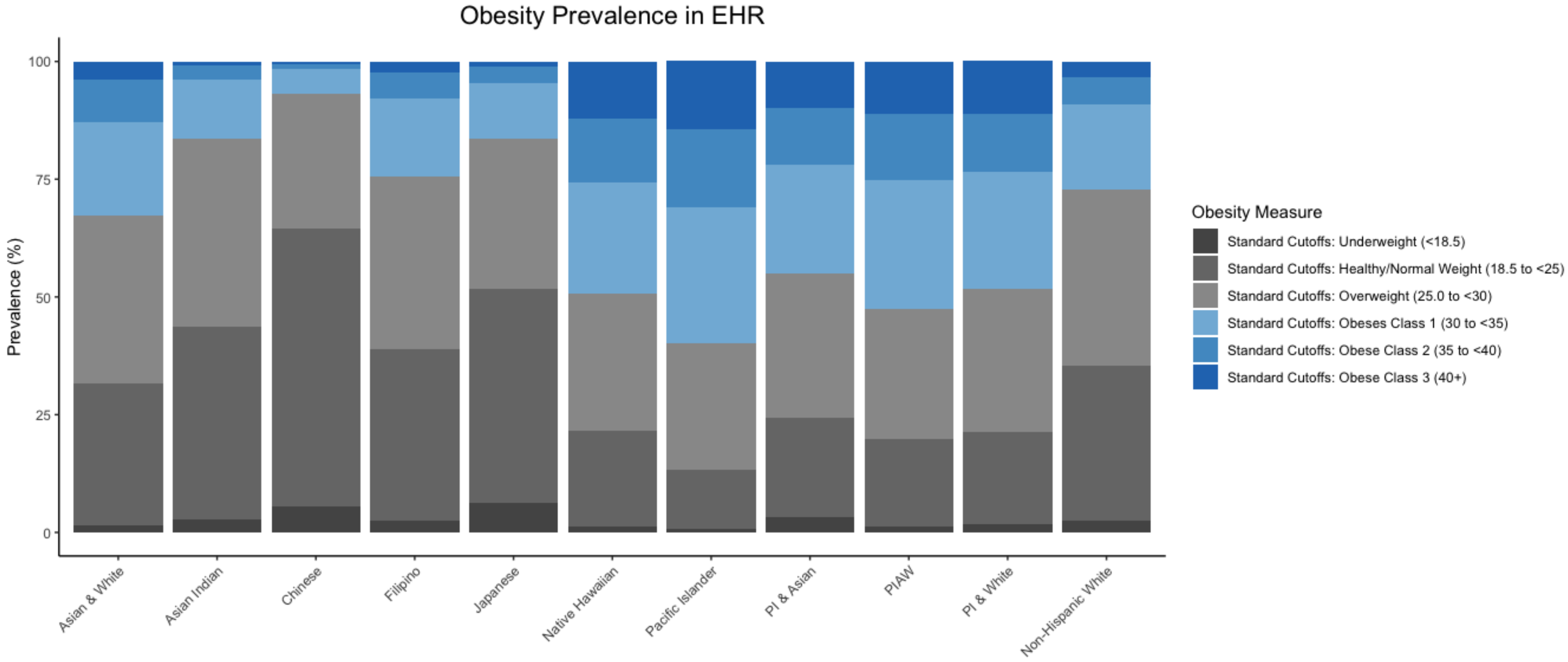

Note. Asian-specific BMI cutoffs are 27.5 kg/m^{2}; these cutoffs were applied to Asian Indian, Chinese, Filipino, and Japanese people only. "Pacific Islander" includes populous groups such as CHamorro/CHamoru, Fijian, Marshallese, Samoan, Tongan, and Tahitian..

© 2024 Bacong AM et al. *JAMA Network Open*.
