# Supplementary material for: Obesity Disparities Among Adult Single-Race and Multiracial Asian and Pacific Islander Populations
Source: JAMA Netw Open. 2024 Mar 19;7(3):e240734. doi: 10.1001/jamanetworkopen.2024.0734 (PMC10951735; doi:10.1001/jamanetworkopen.2024.0734)
Supplement: Supplement 2. — Data Sharing Statement [file jamanetwopen-e240734-s002.pdf]

Data Sharing Statement

Bacong. Obesity Disparities Among Adult Single-Race and Multiracial Asian and Pacific Islander Populations. *JAMA Netw Open*. Published March 19, 2024.
doi:10.1001/jamanetworkopen.2024.0734

Data
Data available: No

Additional Information
Explanation for why data not available: Data were derived from electronic health records from two large healthcare systems. In an effort protect patient identities, data are not publicly available. Should interested investigators desire to analyze the data, they can be made available upon request to the corresponding author.
